# In Memory of the Virologist Jianguo Wu, 1957–2022

**DOI:** 10.3390/v15081754

**Published:** 2023-08-17

**Authors:** Ge Yang, Zhaoyang Yue, Pan Pan, Yongkui Li

**Affiliations:** 1 Foshan Institute of Medical Microbiology, Foshan 528315, China; 2 Institute of Medical Microbiology, Jinan University, Guangzhou 510632, China; 3 Laboratory of Viral Pathogenesis & Infection Prevention and Control (Jinan University), Ministry of Education, Guangzhou 510632, China; 4 The First Affiliated Hospital of Jinan University, Guangzhou 510632, China

## 1. Biographical Introduction

It is with deep sorrow that we mourn the passing of the virologist Professor Jianguo Wu. He devoted his life to the research of viruses, and was still working until the end of his life. His death is a great loss to the international virology community. Here, we summarize his significant contributions to the development of virology, the construction of scientific research platforms, and social services in epidemic prevention and control. Through commemorating our beloved virologist, we are inspired to continue to work hard in his spirit of persistent struggle against viruses.

Professor Jianguo Wu was born in Jishui County, Ji’an, Jiangxi Province, China. From 1978 to 1985, he studied at Wuhan University and engaged in virology research. He went to the United States in 1989 and obtained his Ph.D. in biochemistry from the University of Idaho in 1992. From 1993 to 1996, he engaged in postdoctoral research in molecular biology at Princeton University, and he worked here as a research fellow for another 3 years. In 1999, he resolutely gave up the preferential treatment in the United States and went back to his motherland, China. He worked diligently at Wuhan University for more than 20 years. After retiring, he continued to engage in teaching and research at Jinan University, and also made remarkable achievements there. As soon as the COVID-19 pandemic broke out, he devoted himself to work on SARS-CoV-2 testing and research until the end of his life.

## 2. Academic Achievements

Professor Wu conducted virology research with an emphasis on elucidating the molecular mechanisms of infection, immunity, and the pathogenesis of important human viruses, and the molecular epidemiology of newly emerging viruses. He was devoted to the study of the pathogenesis of human hepatitis B virus (HBV) and hepatitis C virus (HCV). He revealed several important functions of hepatitis B virus x protein (HBx), like its roles in cell apoptosis, transformation, and tumorigenesis [1,2,3]. The association between HBV replication and innate immunity was investigated by his team [4]. He reported several host proteins that could suppress HBV replication [5,6,7,8,9], and found that HBV evolved a series of strategies to evade host immunity, consequently facilitating viral replication [5,10,11,12,13,14,15]. The role of HBV in promoting hepatocellular carcinoma development was studied and characterized by Prof. Wu too [16,17]. In studies of HCV, he revealed the functions of the HCV NS2 protein, NS3 protein, NS5A protein, and the envelope protein E2 in the pathogenesis of viral infection [18,19,20,21,22,23]. He studied the cross talk between HCV and human immunodeficiency virus 1 (HIV-1) and revealed that these two viruses could promote each other’s replication in different ways [24,25,26]. 

The influenza virus has been endangering the life and health of human beings for a long time. Professor Wu was also committed to investigating the epidemiological characteristics of influenza viruses [27], exploring their pathogenesis [28,29] and developing new detection methods [30]. He provided several potential drugs that could effectively inhibit the infection of influenza virus [31,32,33,34,35], and also developed an effective new vaccine [36].

Enteroviruses, particularly Enterovirus 71 viruses, always attracted Prof. Wu’s research interest. He made great achievements in studies about the epidemiological characteristics [37,38] and molecular mechanisms [39,40,41,42,43,44] of EV71. He also discovered some potential drugs against EV71 infection [45]. In particular, he made a breakthrough in the study of EV71-induced inflammatory response, in which the EV71 3D protein contributed to the activation of the NLRP3 inflammasome [46]. 

After retiring from Wuhan University, Professor Wu’s focus shifted to the Pearl River Delta area in China, where Zika and Dengue, of flavivirus, are the more prevalent viruses in the region. He reported the molecular epidemiology and molecular mechanisms of the pathogenesis of Dengue virus [47], and revealed that infection with this virus could induce tissue injury and vascular leakage through its M and NS1 proteins [48,49,50]. Meanwhile, the regulatory mechanism of Zika virus infection in humans was also well elucidated by his team [51,52], especially the inflammation-promoting role of the Zika NS1 protein [53].

The outbreak of SARS and COVID-19 brought great harm to society, and Prof. Wu conducted in-depth and systematic research on SARS-CoV and SARS-CoV-2 [54,55]. Their pathogenesis and epidemiology were identified by his team through an isolation of the virus from a SARS patient [56]. He also revealed the molecular mechanisms of the pathogenesis of SARS-CoV, such as the stimulation of cyclooxygenase-2 expression by spike proteins [57] and the activation of interleukin-6 (IL-6) and cyclooxygenase-2 (COX-2) expression by the viral nucleocapsid protein [58,59]. Due to the good foundation of SARS-CoV research, Professor Wu carried out deeper studies on SARS-CoV-2’s epidemiological characteristics, pathogenic molecular mechanisms, and detection methods. He and his team revealed that the SARS-CoV-2 nucleocapsid protein plays important roles in the repression of IFN-β [60], activation of the NLRP3 inflammasome [61], regulation of apoptosis [62], and the induction of acute kidney injury [63]. He reported that HIF-1α could promote SARS-CoV-2 infection and deepen inflammatory responses [64]. A robust and visual detection of SARS-CoV-2 and emerging variants called loop-mediated isothermal amplification (LAMP) was also developed by Prof. Wu’s team [65]. 

In addition to the studies on the viruses mentioned above, Wu’s team also explored the molecular pathogenesis of viruses including Epstein–Barr virus (EBV) [66], HIV-1 [67], mumps virus [68], Borna disease virus [69,70], and so on. In short, Wu devoted his life to virology research and made great contributions to the development of the field.

## 3. Construction of Scientific Research Platform

**Figure 1:**
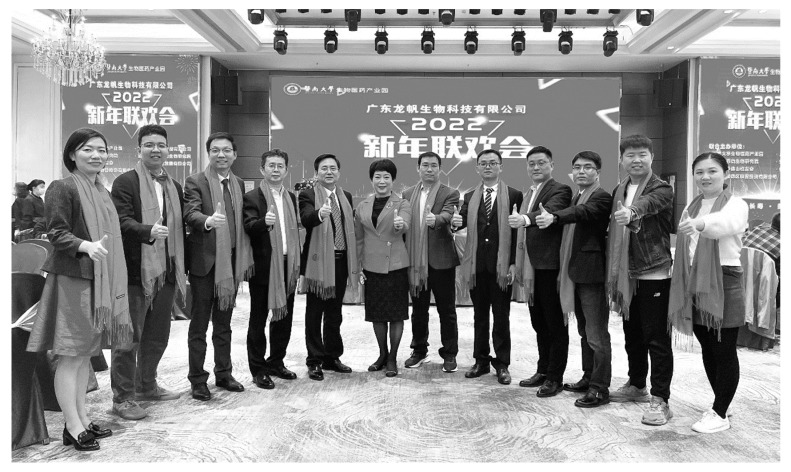
Jianguo Wu and his team at the Institute of Medical Microbiology, Jinan University: Yuanyuan Duan, Heng Xiao, Zhen Luo, Xulin Chen, Jianguo Wu, Qiuping Tan, Qiwei Zhang, Yongkui Li, Jun Chen, Yang Yu, Jinbiao Liu, and Xin Chen.

As a national high-level talent introduction, Professor Wu’s scientific research career was supported by governments at all levels. He presided over more than 30 scientific research projects, including major national science and technology projects, 973 national program projects, and key projects of the National Natural Science Foundation of China. When returning to China and taking up his post at Wuhan University, he devoted himself to the establishment of the State Key Laboratory of Virology (SKLV), played an extremely important role in the establishment of the SKLV, and worked as its director from 2006 to 2016. After retiring, Prof. Wu continued to shine in the virology field. He worked as the president of the Institute of Medical Microbiology at Jinan University (Figure 1). He and his team successively established the Key Laboratory of Virology in Guangzhou and the Key Laboratory of Virology in Guangdong Province at Jinan University. In order to facilitate industrial transformation, Prof. Wu established the Biomedical Industry Park of Jinan University and the Foshan Institute of Medical Microbiology with support from the Foshan Municipal government and the Shunde District government, respectively. 

Under the leadership of Prof. Wu, these research platforms have produced good scientific output, served local education and scientific research, and contributed to local economic development.

## 4. Social Contribution and Personal Honor

**Figure 2:**
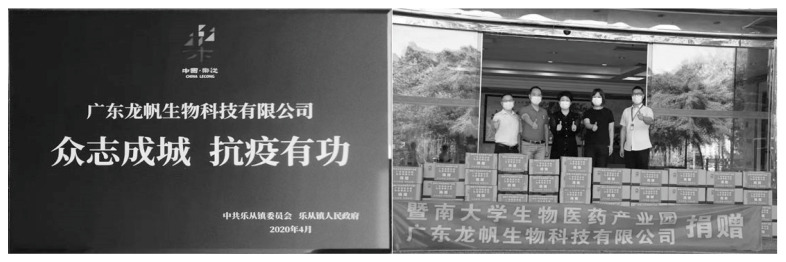
(**Left**) the local government awarded Wu’s company a medal for meritorious efforts in combating the epidemic. (**Right**) Wu’s company donated epidemic prevention materials.

Throughout his career, Prof. Wu made outstanding achievements. He published over 230 papers in scientific journals, and he was selected as one of the most cited Chinese Researchers in 2021 by Elsevier. He was also granted 32 authorized national invention patents. During the COVID-19 pandemic, Professor Wu’s company (Longfan Biotechnology Co., Ltd.) donated free disinfection and sterilization products to the community, which solved the problem of supply shortage. This selfless dedication received praise from the local government (Figure 2). 

In recognition of his outstanding contribution, he received 30 awards in the last 25 years. They included the first prize for Natural Science in Hubei Province, the second prize for Natural Science in Yunnan Province, the second prize for life chemistry research in Yaoming Kant, the first prize for outstanding team in the implementation of national science and technology plan, and the first prize for excellent academic papers in Natural Science in Hubei Province. His honors also include a special allowance provided by the government of the State Council, Excellent Foreign Experts of the People’s Government of Hubei Province, Top Ten Young and Middle-Aged Experts with Outstanding Contributions in Hubei Province, Advanced Individuals of New Overseas Chinese Entrepreneurship in Hubei Province, etc. 

## 5. Conclusions

In short, Prof. Wu made indelible contributions to education, virology research, and the welfare of society. He achieved many great things in his short life. We pay tribute to our beloved teacher with this article to express our deep thoughts for him.

## References

[1] Wei W., Huang W., Pan Y., Zhu F., Wu J. (2006). Functional switch of viral protein HBx on cell apoptosis, transformation, and tumorigenesis in association with oncoprotein Ras. Cancer Lett..

[2] Xia L.M., Huang W.J., Wu J.G., Yang Y.B., Zhang Q., Zhou Z.Z., Zhu H.F., Lei P., Shen G.X., Tian D.A. (2009). HBx protein induces expression of MIG and increases migration of leukocytes through activation of NF-kappaB. Virology.

[3] Mu Y., Yu Y., Yue X., Musarat I., Gong R., Zhu C., Liu Y., Liu F., Zhu Y., Wu J. (2011). The X protein of HBV induces HIV-1 long terminal repeat transcription by enhancing the binding of C/EBPbeta and CREB1/2 regulatory proteins to the long terminal repeat of HIV-1. Virus Res..

[4] Yang G., Wan P., Zhang Y., Tan Q., Qudus M.S., Yue Z., Luo W., Zhang W., Ouyang J., Li Y. (2022). Innate Immunity, Inflammation, and Intervention in HBV Infection. Viruses.

[5] Yang H., Zhou Y., Mo J., Xiang Q., Qin M., Liu W., Shang J., Yang Q., Xu W., Yang G. (2020). SOX9 represses hepatitis B virus replication through binding to HBV EnhII/Cp and inhibiting the promoter activity. Antiviral Res..

[6] Yang Q., Zhang Q., Zhang X., You L., Wang W., Liu W., Han Y., Ma C., Xu W., Chen J. (2019). HoxA10 Facilitates SHP-1-Catalyzed Dephosphorylation of p38 MAPK/STAT3 To Repress Hepatitis B Virus Replication by a Feedback Regulatory Mechanism. J. Virol..

[7] Xu W., Ma C., Zhang Q., Zhao R., Hu D., Zhang X., Chen J., Liu F., Wu K., Liu Y. (2018). PJA1 Coordinates with the SMC5/6 Complex To Restrict DNA Viruses and Episomal Genes in an Interferon-Independent Manner. J. Virol..

[8] Xia C., Chen Y.C., Gong H., Zeng W., Vu G.P., Trang P., Lu S., Wu J., Liu F. (2013). Inhibition of hepatitis B virus gene expression and replication by ribonuclease P. Mol. Ther..

[9] Xu Y., Yang Y., Cai Y., Liu F., Liu Y., Zhu Y., Wu J. (2011). The X protein of hepatitis B virus activates hepatoma cell proliferation through repressing melanoma inhibitory activity 2 gene. Biochem. Biophys. Res. Commun..

[10] Chen J., Xu W., Chen Y., Xie X., Zhang Y., Ma C., Yang Q., Han Y., Zhu C., Xiong Y. (2017). Matrix Metalloproteinase 9 Facilitates Hepatitis B Virus Replication through Binding with Type I Interferon (IFN) Receptor 1 to Repress IFN/JAK/STAT Signaling. J. Virol..

[11] Yu Y., Wan P., Cao Y., Zhang W., Chen J., Tan L., Wang Y., Sun Z., Zhang Q., Wan Y. (2017). Hepatitis B Virus e Antigen Activates the Suppressor of Cytokine Signaling 2 to Repress Interferon Action. Sci. Rep..

[12] Bai L., Zhang W., Tan L., Yang H., Ge M., Zhu C., Zhang R., Cao Y., Chen J., Luo Z. (2015). Hepatitis B virus hijacks CTHRC1 to evade host immunity and maintain replication. J. Mol. Cell. Biol..

[13] Shang J., Zheng Y., Guo X., Mo J., Xie X., Xiong Y., Liu Y., Wu K., Wu J. (2015). Hepatitis B virus replication and sex-determining region Y box 4 production are tightly controlled by a novel positive feedback mechanism. Sci. Rep..

[14] Cao Y., Zhang R., Zhang W., Zhu C., Yu Y., Song Y., Wang Q., Bai L., Liu Y., Wu K. (2014). IL-27, a cytokine, and IFN-lambda1, a type III IFN, are coordinated to regulate virus replication through type I IFN. J. Immunol..

[15] Yu Y., Gong R., Mu Y., Chen Y., Zhu C., Sun Z., Chen M., Liu Y., Zhu Y., Wu J. (2011). Hepatitis B virus induces a novel inflammation network involving three inflammatory factors, IL-29, IL-8, and cyclooxygenase-2. J. Immunol..

[16] Liu Z., Dai X., Wang T., Zhang C., Zhang W., Zhang W., Zhang Q., Wu K., Liu F., Liu Y. (2017). Hepatitis B virus PreS1 facilitates hepatocellular carcinoma development by promoting appearance and self-renewal of liver cancer stem cells. Cancer Lett..

[17] Zhang R., Cao Y., Bai L., Zhu C., Li R., He H., Liu Y., Wu K., Liu F., Wu J. (2015). The collagen triple helix repeat containing 1 facilitates hepatitis B virus-associated hepatocellular carcinoma progression by regulating multiple cellular factors and signal cascades. Mol. Carcinog..

[18] Yang X.J., Liu J., Ye L., Liao Q.J., Wu J.G., Gao J.R., She Y.L., Wu Z.H., Ye L.B. (2006). HCV NS2 protein inhibits cell proliferation and induces cell cycle arrest in the S-phase in mammalian cells through down-regulation of cyclin A expression. Virus Res..

[19] Lu L., Wei L., Peng G., Mu Y., Wu K., Kang L., Yan X., Zhu Y., Wu J. (2008). NS3 protein of hepatitis C virus regulates cyclooxygenase-2 expression through multiple signaling pathways. Virology.

[20] Lu L., Zhang Q., Wu K., Chen X., Zheng Y., Zhu C., Wu J. (2015). Hepatitis C virus NS3 protein enhances cancer cell invasion by activating matrix metalloproteinase-9 and cyclooxygenase-2 through ERK/p38/NF-kappaB signal cascade. Cancer Lett..

[21] Zhang X., Wang T., Dai X., Zhang Y., Jiang H., Zhang Q., Liu F., Wu K., Liu Y., Zhou H. (2016). Golgi protein 73 facilitates the interaction of hepatitis C virus NS5A with apolipoprotein E to promote viral particle secretion. Biochem. Biophys. Res. Commun..

[22] Pan Y., Wei W., Kang L., Wang Z., Fang J., Zhu Y., Wu J. (2007). NS5A protein of HCV enhances HBV replication and resistance to interferon response. Biochem. Biophys. Res. Commun..

[23] Li P., Wan Q., Feng Y., Liu M., Wu J., Chen X., Zhang X.L. (2007). Engineering of N-glycosylation of hepatitis C virus envelope protein E2 enhances T cell responses for DNA immunization. Vaccine.

[24] Kang L., Luo Z., Li Y., Zhang W., Sun W., Li W., Chen Y., Liu F., Xia X., Zhu Y. (2012). Association of Vpu with hepatitis C virus NS3/4A stimulates transcription of type 1 human immunodeficiency virus. Virus Res..

[25] Yang Y., Wu J., Lu Y. (2010). Mechanism of HIV-1-TAT induction of interleukin-1beta from human monocytes: Involvement of the phospholipase C/protein kinase C signaling cascade. J. Med. Virol..

[26] Qu J., Yang Z., Zhang Q., Liu W., Li Y., Ding Q., Liu F., Liu Y., Pan Z., He B. (2011). Human immunodeficiency virus-1 Rev protein activates hepatitis C virus gene expression by directly targeting the HCV 5′-untranslated region. FEBS Lett..

[27] Mukhtar M.M., Rasool S.T., Song D., Zhu C., Hao Q., Zhu Y., Wu J. (2007). Origin of highly pathogenic H5N1 avian influenza virus in China and genetic characterization of donor and recipient viruses. J. Gen. Virol..

[28] Li W., Liu Y., Mukhtar M.M., Gong R., Pan Y., Rasool S.T., Gao Y., Kang L., Hao Q., Peng G. (2008). Activation of interleukin-32 pro-inflammatory pathway in response to influenza A virus infection. PLoS ONE.

[29] Yang Y., Zengel J., Sun M., Sleeman K., Timani K.A., Aligo J., Rota P., Wu J., He B. (2015). Regulation of Viral RNA Synthesis by the V Protein of Parainfluenza Virus 5. J. Virol..

[30] Yang Z., Mao G., Liu Y., Chen Y.C., Liu C., Luo J., Li X., Zen K., Pang Y., Wu J. (2013). Detection of the pandemic H1N1/2009 influenza A virus by a highly sensitive quantitative real-time reverse-transcription polymerase chain reaction assay. Virol. Sin..

[31] Mukhtar M.M., Li S., Li W., Wan T., Mu Y., Wei W., Kang L., Rasool S.T., Xiao Y., Zhu Y. (2009). Single-chain intracellular antibodies inhibit influenza virus replication by disrupting interaction of proteins involved in viral replication and transcription. Int. J. Biochem. Cell Biol..

[32] An L., Liu R., Tang W., Wu J.G., Chen X. (2014). Screening and identification of inhibitors against influenza A virus from a US drug collection of 1280 drugs. Antiviral Res..

[33] Zhang W., Chen S.T., He Q.Y., Huang L.Q., Li X., Lai X.P., Zhan S.F., Huang H.T., Liu X.H., Wu J. (2018). Asprellcosides B of Ilex asprella Inhibits Influenza A Virus Infection by Blocking the Hemagglutinin- Mediated Membrane Fusion. Front. Microbiol..

[34] Chen S., Liu G., Chen J., Hu A., Zhang L., Sun W., Tang W., Liu C., Zhang H., Ke C. (2019). Ponatinib Protects Mice from Lethal Influenza Infection by Suppressing Cytokine Storm. Front. Immunol..

[35] Wan P., Zhang S., Ruan Z., Liu X., Yang G., Jia Y., Li Y., Pan P., Wang W., Li G. (2022). AP-1 signaling pathway promotes pro-IL-1beta transcription to facilitate NLRP3 inflammasome activation upon influenza A virus infection. Virulence.

[36] Pei Z., Jiang X., Yang Z., Ren X., Gong H., Reeves M., Sheng J., Wang Y., Pan Z., Liu F. (2015). Oral Delivery of a Novel Attenuated Salmonella Vaccine Expressing Influenza A Virus Proteins Protects Mice against H5N1 and H1N1 Viral Infection. PLoS ONE.

[37] Liu W., Wu S., Xiong Y., Li T., Wen Z., Yan M., Qin K., Liu Y., Wu J. (2014). Co-circulation and genomic recombination of coxsackievirus A16 and enterovirus 71 during a large outbreak of hand, foot, and mouth disease in Central China. PLoS ONE.

[38] Liu M.Y., Liu J., Lai W., Luo J., Liu Y., Vu G.P., Yang Z., Trang P., Li H., Wu J. (2016). Characterization of enterovirus 71 infection and associated outbreak of Hand, Foot, and Mouth Disease in Shawo of China in 2012. Sci. Rep..

[39] Song Y., Cheng X., Yang X., Zhao R., Wang P., Han Y., Luo Z., Cao Y., Zhu C., Xiong Y. (2015). Early growth response-1 facilitates enterovirus 71 replication by direct binding to the viral genome RNA. Int. J. Biochem. Cell Biol..

[40] Jin J., Wang W., Ai S., Liu W., Song Y., Luo Z., Zhang Q., Wu K., Liu Y., Wu J. (2019). Enterovirus 71 Represses Interleukin Enhancer-Binding Factor 2 Production and Nucleus Translocation to Antagonize ILF2 Antiviral Effects. Viruses.

[41] You L., Chen J., Liu W., Xiang Q., Luo Z., Wang W., Xu W., Wu K., Zhang Q., Liu Y. (2020). Enterovirus 71 induces neural cell apoptosis and autophagy through promoting ACOX1 downregulation and ROS generation. Virulence.

[42] Xiang Q., Wan P., Yang G., Huang S., Qin M., Yang H., Luo Z., Wu K., Wu J. (2020). Beclin1 Binds to Enterovirus 71 3D Protein to Promote the Virus Replication. Viruses.

[43] Luo Z., Ge M., Chen J., Geng Q., Tian M., Qiao Z., Bai L., Zhang Q., Zhu C., Xiong Y. (2017). HRS plays an important role for TLR7 signaling to orchestrate inflammation and innate immunity upon EV71 infection. PLoS Pathog..

[44] Han Y., Wang L., Cui J., Song Y., Luo Z., Chen J., Xiong Y., Zhang Q., Liu F., Ho W. (2016). SIRT1 inhibits EV71 genome replication and RNA translation by interfering with the viral polymerase and 5′UTR RNA. J. Cell Sci..

[45] Zhang W., Tao J., Yang X., Yang Z., Zhang L., Liu H., Wu K., Wu J. (2014). Antiviral effects of two Ganoderma lucidum triterpenoids against enterovirus 71 infection. Biochem. Biophys. Res. Commun..

[46] Wang W., Xiao F., Wan P., Pan P., Zhang Y., Liu F., Wu K., Liu Y., Wu J. (2017). EV71 3D Protein Binds with NLRP3 and Enhances the Assembly of Inflammasome Complex. PLoS Pathog..

[47] Li G., Pan P., He Q., Kong X., Wu K., Zhang W., Liu Y., Huang H., Liu J., Zhang Z. (2017). Molecular epidemiology demonstrates that imported and local strains circulated during the 2014 dengue outbreak in Guangzhou, China. Virol. Sin..

[48] Pan P., Zhang Q., Liu W., Wang W., Lao Z., Zhang W., Shen M., Wan P., Xiao F., Liu F. (2019). Dengue Virus M Protein Promotes NLRP3 Inflammasome Activation To Induce Vascular Leakage in Mice. J. Virol..

[49] Pan P., Zhang Q., Liu W., Wang W., Yu Z., Lao Z., Zhang W., Shen M., Wan P., Xiao F. (2019). Dengue Virus Infection Activates Interleukin-1beta to Induce Tissue Injury and Vascular Leakage. Front. Microbiol..

[50] Pan P., Li G., Shen M., Yu Z., Ge W., Lao Z., Fan Y., Chen K., Ding Z., Wang W. (2021). DENV NS1 and MMP-9 cooperate to induce vascular leakage by altering endothelial cell adhesion and tight junction. PLoS Pathog..

[51] Li A., Wang W., Wang Y., Chen K., Xiao F., Hu D., Hui L., Liu W., Feng Y., Li G. (2020). NS5 Conservative Site Is Required for Zika Virus to Restrict the RIG-I Signaling. Front. Immunol..

[52] Wang Y., Li Q., Hu D., Gao D., Wang W., Wu K., Wu J. (2021). USP38 Inhibits Zika Virus Infection by Removing Envelope Protein Ubiquitination. Viruses.

[53] Wang W., Li G., De W., Luo Z., Pan P., Tian M., Wang Y., Xiao F., Li A., Wu K. (2018). Zika virus infection induces host inflammatory responses by facilitating NLRP3 inflammasome assembly and interleukin-1beta secretion. Nat. Commun..

[54] Li G., Fan Y., Lai Y., Han T., Li Z., Zhou P., Pan P., Wang W., Hu D., Liu X. (2020). Coronavirus infections and immune responses. J. Med. Virol..

[55] Qudus M.S., Tian M., Sirajuddin S., Liu S., Afaq U., Wali M., Liu J., Pan P., Luo Z., Zhang Q. (2023). The roles of critical pro-inflammatory cytokines in the drive of cytokine storm during SARS-CoV-2 infection. J. Med. Virol..

[56] Zhu Y., Liu M., Zhao W., Zhang J., Zhang X., Wang K., Gu C., Wu K., Li Y., Zheng C. (2005). Isolation of virus from a SARS patient and genome-wide analysis of genetic mutations related to pathogenesis and epidemiology from 47 SARS-CoV isolates. Virus Genes.

[57] Abdel-Latif M.S. (2015). Plasma Levels of Matrix Metalloproteinase (MMP)-2, MMP-9 and Tumor Necrosis Factor-alpha in Chronic Hepatitis C Virus Patients. Open Microbiol. J..

[58] Zhang X., Wu K., Wang D., Yue X., Song D., Zhu Y., Wu J. (2007). Nucleocapsid protein of SARS-CoV activates interleukin-6 expression through cellular transcription factor NF-kappaB. Virology.

[59] Yan X., Hao Q., Mu Y., Timani K.A., Ye L., Zhu Y., Wu J. (2006). Nucleocapsid protein of SARS-CoV activates the expression of cyclooxygenase-2 by binding directly to regulatory elements for nuclear factor-kappa B and CCAAT/enhancer binding protein. Int. J. Biochem. Cell Biol..

[60] Chen K., Xiao F., Hu D., Ge W., Tian M., Wang W., Pan P., Wu K., Wu J. (2020). SARS-CoV-2 Nucleocapsid Protein Interacts with RIG-I and Represses RIG-Mediated IFN-beta Production. Viruses.

[61] Pan P., Shen M., Yu Z., Ge W., Chen K., Tian M., Xiao F., Wang Z., Wang J., Jia Y. (2021). SARS-CoV-2 N protein promotes NLRP3 inflammasome activation to induce hyperinflammation. Nat. Commun..

[62] Pan P., Ge W., Lei Z., Luo W., Liu Y., Guan Z., Chen L., Yu Z., Shen M., Hu D. (2023). SARS-CoV-2 N protein enhances the anti-apoptotic activity of MCL-1 to promote viral replication. Signal Transduct. Target Ther..

[63] Wang W., Chen J., Hu D., Pan P., Liang L., Wu W., Tang Y., Huang X.R., Yu X., Wu J. (2022). SARS-CoV-2 N Protein Induces Acute Kidney Injury via Smad3-Dependent G1 Cell Cycle Arrest Mechanism. Adv. Sci..

[64] Tian M., Liu W., Li X., Zhao P., Shereen M.A., Zhu C., Huang S., Liu S., Yu X., Yue M. (2021). HIF-1alpha promotes SARS-CoV-2 infection and aggravates inflammatory responses to COVID-19. Signal Transduct. Target Ther..

[65] Luo Z., Ye C., Xiao H., Yin J., Liang Y., Ruan Z., Luo D., Gao D., Tan Q., Li Y. (2022). Optimization of loop-mediated isothermal amplification (LAMP) assay for robust visualization in SARS-CoV-2 and emerging variants diagnosis. Chem. Eng. Sci..

[66] Zhang W., Han D., Wan P., Pan P., Cao Y., Liu Y., Wu K., Wu J. (2016). ERK/c-Jun Recruits Tet1 to Induce Zta Expression and Epstein-Barr Virus Reactivation through DNA Demethylation. Sci. Rep..

[67] Yang Y., Liu W., Hu D., Su R., Ji M., Huang Y., Shereen M.A., Xu X., Luo Z., Zhang Q. (2020). HIV-1 Nef Interacts with LMP7 to Attenuate Immunoproteasome Formation and Major Histocompatibility Complex Class I Antigen Presentation. mBio.

[68] Xu P., Huang Z., Gao X., Michel F.J., Hirsch G., Hogan R.J., Sakamoto K., Ho W., Wu J., He B. (2013). Infection of mice, ferrets, and rhesus macaques with a clinical mumps virus isolate. J. Virol..

[69] Peng G., Zhang F., Zhang Q., Wu K., Zhu F., Wu J. (2007). Borna disease virus P protein inhibits nitric oxide synthase gene expression in astrocytes. Virology.

[70] Peng G., Yan Y., Zhu C., Wang S., Yan X., Lu L., Li W., Hu J., Wei W., Mu Y. (2008). Borna disease virus P protein affects neural transmission through interactions with gamma-aminobutyric acid receptor-associated protein. J. Virol..

